# The Effect and Mechanism of Growth Hormone Replacement on Cognitive Function in Rats with Traumatic Brain Injury

**DOI:** 10.1371/journal.pone.0108518

**Published:** 2014-09-30

**Authors:** Hao Zhang, Mengqi Han, Xiaonian Zhang, Xinting Sun, Feng Ling

**Affiliations:** 1 China Rehabilitation Research Center, Capital Medical University, Beijing, China; 2 Beijing Jishuitan Hospital, Beijing, China; 3 Beijing Xuanwu Hospital, Capital Medical University, Beijing, China; Federal University of Rio de Janeiro, Brazil

## Abstract

**Objective:**

The effects of growth hormone on cognitive dysfunction were observed in a controlled cortical impact (CCI) rat model and the underlying mechanism was explored.

**Method:**

Three-month-old male SD rats were randomly divided into sham (n = 10), control (n = 10), and CCI groups (n = 40) The parameters were set as follows: striking speed, 3.5 m/s; impact depth, 1.5 mm; and dwell time, 400 msec. Eight and ten weeks post-injury, the GH levels were measured the water maze test and novel object recognition test were performed. CCI rats were divided into normal and decreased GH groups, and further randomly divided into two sub-groups (rhGH treatment and saline vehicle groups). All rats were tested for SYN, BDNF, and TrkB mRNA in the prefrontal cortex and hippocampus by RT-PCR.

**Results:**

CCI rats 8 weeks post-injury had cognitive dysfunction regardless of the GH level (P<0.05). rhGH treatment improved cognitive function in CCI rats. There was a positive correlation between the expression of prefrontal BDNF and SYN mRNA in CCI rats after rhGH therapy and the water maze test score (r = 0.773 and 0.534, respectively; P<0.05). Furthermore, the expression of BDNF, TrkB, and SYN mRNA in the hippocampus was negatively correlated with the water maze test score (r = 0.602, 0.773, 0.672, and 0.783, respectively; P<0.05). There was a difference in the expression of hippocampal and prefrontal BDNF, TrkB, and SYN mRNA (P<0.05)

**Conclusion:**

rhGH treatment had a positive effect on cognitive function, which was more evident in GH-deficient rats. The increased expression of hippocampal and prefrontal BDNF and TrkB mRNA is implicated in rhGH therapy to improve cognitive function. Changes in the expression of hippocampal SYN mRNA following rhGH therapy may also play a role in improving cognitive function.

## Introduction

Traumatic brain injury (TBI) is a medical condition with high morbidity worldwide, and leading mortality and disability rates [1]. Cognitive impairment is one of the most common symptoms in patients with moderate-to-severe TBI. Patients with varying degrees of TBI may develop cognitive impairment, causing a significant impact on daily life, which impedes a patient returning to society. Endocrine dysfunction after a TBI may be due to primary or secondary lesions in the hypothalamus and pituitary. Any degree of TBI can cause varying changes in pituitary hormone levels [2], the most prominent of which is growth hormone (GH) deficiency. GH is important to the development of the central nervous system, especially in promoting the maturation of the brain, myelin formation, glial cell differentiation, and cognitive function. The relationship between the GH level and cognitive function has been studied in Alzheimer’s disease (AD) [3]–[5] and patients with growth hormone deficiency (GHD) [6], [7]. The relationship among TBI, GH, and recombinant human growth hormone (rhGH) therapy has been the focus of clinical epidemiologic studies [8]; however, there are few experimental studies and the underlying mechanism is unclear.

In 1986 the genetic synthesis of rhGH, which is identical to the natural structure and function of human GH, was reported for the treatment of GHD [9]. There are also some reports of TBI patients with GH deficiency who had neurologic improvement and quality of life after being treated with rhGH [10]–[12]. The lesion sites in TBI primarily involve the frontal and temporal lobes, basal ganglia, and hippocampus, all of which can cause cognitive disorders. Among the cranial structures affected by a TBI, the hippocampus is the most common site for lesions.

A controlled cortical impact (CCI) is considered to be an accurate TBI model, which can control the cerebral hemodynamic parameters, including striking speed, dwell time, impact depth, and site. Therefore, the CCI model can replicate different degrees of brain damage with greater reproducibility and less affected by individual differences [13], [14]. Rats have low price, abundant source and pure strains than other animals, model making procedure is simple, the survival rate is high; and rats have the similar cerebral vascular anatomy and physiology to humans, so easy to study anatomy; and social ethics are more likely to be accepted than other large mammals, so choose SD rats to make the TBI model. The mechanism implicated in long-term GH deficiency caused by CCI is ill-defined, and cannot explain the neuroprotective effects in TBI settings. Currently, the neuroendocrine changes associated with cognitive impairment in patients with TBI have become a research hotspot.

Brain-derived neurotrophic factor (BDNF) is a class of secreted proteins that affect nervous system plasticity and is closely associated with synaptic activity. The synaptic function of all neurotrophins is adjusted by Trk receptors via binding. BDNF does not only maintain neuronal survival, but also has an extremely important impact on synaptic development [15]. At present, controversy exists regarding the effect of BDNF and TrkB on hippocampal neurons and cognitive function after TBI. Miladi et al. [16] reported that cognitive impairment in rats addicted to morphine is likely to be mediated through the TrkB molecular pathway. Synaptophysin (SYN) is the phosphorylated protein connected with the synaptic vesicle, which is the specific protein on the synaptic vesicle membrane involved in synaptic vesicle import, transit, and neurotransmitter release [17]. Teny et al. [18] reported that the immunoreactivity of SYN is related to the degree of cognitive impairment in patients with AD, and furthermore, hippocampal synaptophysin immunohistochemical staining was significantly reduced in aged rats with memory impairment compared to those with normal memory.

There are few studies that have investigated the effect of rhGH treatment following TBI. Because of limitations in clinical research, our project was focused on the relationship between decreased GH following TBI and cognitive dysfunction. In addition, we investigated the relationships after rhGH treatment from the genetic level to determine the underlying mechanism for the clinical use of rhGH.

## Subjects and Methods

### 1.1 Subjects and groups

All procedures were performed in accordance with the Chinese Capital Medical University Animal Welfare guidelines and approved by the Institutional Animal Care and Use Committee of the Chinese Capital Medical University. Sixty 3-month-old healthy, clean, male SD rats, weighing 275±25 g (purchased from the PLA Academy of the Military Medical Experimental Animal Center), were used in this study. We performed the animal experiments in strict compliance with medical laboratory animal management implementation rules and other relevant laws and regulations. The rearing conditions were as follows: temperature, 21±1°C; light/dark cycle, 12 h/12 h; and illumination time, 0700–1900. Sufficient water and food were given daily before and after surgery; the rats fasted for 8 hours pre-operatively. The rodents were randomly divided into three groups, as follows: normal control group (n = 10); sham group (n = 10); and CCI group (n = 40). Based on the GH level 8 weeks post-surgery, the rats in the CCI group were further divided into two subgroups (CCI-N and CCI-D groups). Each rats was pierced in ear to number, experiments were proceed by number order.

### 1.2 Brain injury model

All of the following animal experiments were carried out in the course of SPF animals laboratory. We used the eCCI-6.3 device (New York University) as previously described [19]. Rats were administered 10% chloral hydrate (0.3 ml/100 g ip), (Chloral hydrate is relatively safe, easy to control dose and no respiratory damage.) then underwent a bilateral frontal craniotomy, and the lesion site was set 3 mm anterior and lateral to the bregma. The diameter of the bone window was ≥5 mm. A CCI impactor was used to cause the cortical injury, and the parameters were set as followed: striking speed, 3.5 m/s; blow depth, 1.5 mm; and dwell time, 400 msec [20]. The whole procedure lasts 15 to 20 min, maintain body temperature all the time, after the surgery, rats were placed on a warm blanket in an hour and then returned to their own cages. The normal control group received no intervention, and the sham group underwent craniotomies without a striking blow [21].

### 1.3 Determination of growth hormone

Tail blood was collected before the CCI, 8 weeks after the CCI, and 10 weeks (rhGH replacement therapy for 2 weeks) after CCI. The volume of blood was 1.5–2.0 ml, and after centrifugation, the upper serum fraction was tested by radioimmunoassay (provided by the Di Ann Medical Testing Center) for GH. The unit of GH was ng/ml.

### 1.4 Administration of recombinant human growth hormone

The rhGH was provided by Changchun Kinsey Pharmaceutical Company [Changchun, China]. The dose was 30 IU/ml. The administration of rhGH was via subcutaneous cervical injection at a dose of 200 µg/kg [22]. The same dose of saline was given to the control group. These injections were administered once a day at 8:00 am [23] for 2 consecutive weeks.

### 1.5 Water maze test

All rats were tested in a water maze at baseline. At 8 and 10 weeks (rhGH replacement therapy for 2 weeks) post-injury, the water maze test was performed (MWM model XR-XM101, Shanghai Xinruan Technology Co., Ltd., Shanghai, China). The water temperature was controlled at 28±1°C, and melanin was added to the water to visualize the underwater platform clearly and reduce the impact on the positioning of underwater structures [23]. For the navigation test, the platform was located in one quadrant and 2 cm below the water surface. The rat was put in the maze facing the maze wall. If the rat could not find the hidden platform within 60 seconds, the rat was guided to the platform by the experimenter, where the rat remained for 10 seconds. During 3 consecutive days, rats were trained 4 times a day in the platform, and the interval of time between the two training session was 15–20 minutes. The latency time was recorded [24]. For the space exploration experiment, followed the navigation test, the rat would be put into the opposite quadrant, and the percentage of total time spent in the original quadrant within 60 seconds was recorded.

### 1.6 Novel object recognition test

Baseline of novel object recognition of all rats were measured one day before the CCI test. Object recognition experiments were repeated in rats at the end of 8 and 10 weeks after CCI (i.e. 2 weeks after rhGH replacement therapy) respectively. The rats were first exposed to the empty arena (60*60*45 cm, made of black PVC, XR-XX117, Shanghai Xinruan Technology Co., Ltd., Shanghai, China) for 5 min (day 0) under bright light, to minimize stress related to a novel environment. After the first hour, the rats were allowed to interact with two identical objects for 5 min. The rats were then re-introduced into the arena for 3 min after the second hour, with one familiar and one novel object this time. The position of the objects remained the same in the arena to remove any spatial memory component from the task. The animals were filmed with a camera (Shanghai Xinruan Technology Co., Ltd., Shanghai, China) and the interaction time(nose at 2 cm or less in the object’s direction)with the novel and familiar objects, and distance traveled(cm) were analyzed with the TopScan2.0 tracking software(Shanghai Xinruan Technology Co., Ltd., Shanghai, China). The above experiments were also recorded by cameras. The time rats explore the old object (object A) and the new one (object B) were recorded by Stopwatch Software (TimeLeft3) respectively, and the discrimination index (DI) were calculated. The formula for is DI = (N−F)/(N+F)×100%. N referes to the time the rats used to explore the new objects. F refers the time the rats to explore the old objects.

### 1.7 Detection of BDNF, TrkB, and SYN mRNA levels by real-time PCR

After the behavioral test, each rat was sacrificed and the brain was removed. The brain tissue was placed on ice; the hippocampus and prefrontal brain tissue were isolated and frozen in liquid nitrogen. The total RNA was extracted for RT- PCR analysis. GADPH was used as the internal reference, and the gene sequence is shown in Table 1. Primers were synthesized by Beijing Reagan Biotechnology Co., Ltd. (Beijing, China). The TL988 PCR instrument detection system [INESA Instrument, Shanghai, China] was used for quantitative RT-PCR analysis, and 5′ -3′ exonuclease activity was amplified by Taq polymerase. The PCR conditions were as follows: 95°C for 2 min; 94°C for 30 s, 54°C fir 30 s, and 72°C for 30 s; and the fluorescence value was read at 72°C. Altogether, there were 45 cycles. The 2^−ΔΔct^ value method was used to calculate the relative ratio of the Ct value with the TL988 PCR instrument software acquisition (PCR software analysis system).

**Table 1:** Real-time PCR Primer Sequence.

Gene size (bp)		Primer
BDNF	Upstream: 5′- ctgcgcccatgaaagaag- 3′Downstream: 5′- attcctccagcagaaaga -3′	291
TrkB	Upstream: 5′- acggagtaccacggctg-3′Downstream: 5′-gcaatcaccaccacggca-3′	299
SYN	Upstream: 5′- ctgcgcccatgaaagaag- 3′Downstream: 5′- attcctccagcagaaaga -3′	188
GAPDH	Upstream: 5′- gctggcattgctctcaatg - 3′Downstream: 5′- cctctctcttgctctcag -3′	163

### 1.8 Statistical analysis

Data were analyzed using SPSS 16.0 software. Ordinal data are expressed as the absolute value and percentage, and measurable data are presented as the mean ± standard deviation (x¯±s). GH values were compared with paired t-tests. The data from the MWM were analyzed by repeated-measures ANOVA, and space exploration data from the same time point were compared among groups using one-way ANOVA, with *post hoc* LSD for the comparison between groups. The latency period was translated into scores for each group (highest = 60 points and lowest = 1 point) corresponding to the time spent. Then, the result was averaged for 3 days, as a result of cognitive function [25]. The data from the novel object recognition test were analyzed by ANOVA test and group comparisons were analyzed by the LSD *post hoc* test. The data from CCI-N and CCI-D were analyzed by paired T-test. Correlations were determined for BDNF, Trk, and SYN mRNA in different parts of the brain and cognitive function test scores the by rank-sum test. When the difference was statistically significant, the LSD test was used. A P<0.05 was considered statistically significant.

## Results

### 2.1 GH levels at different time points

**Figure 1:**
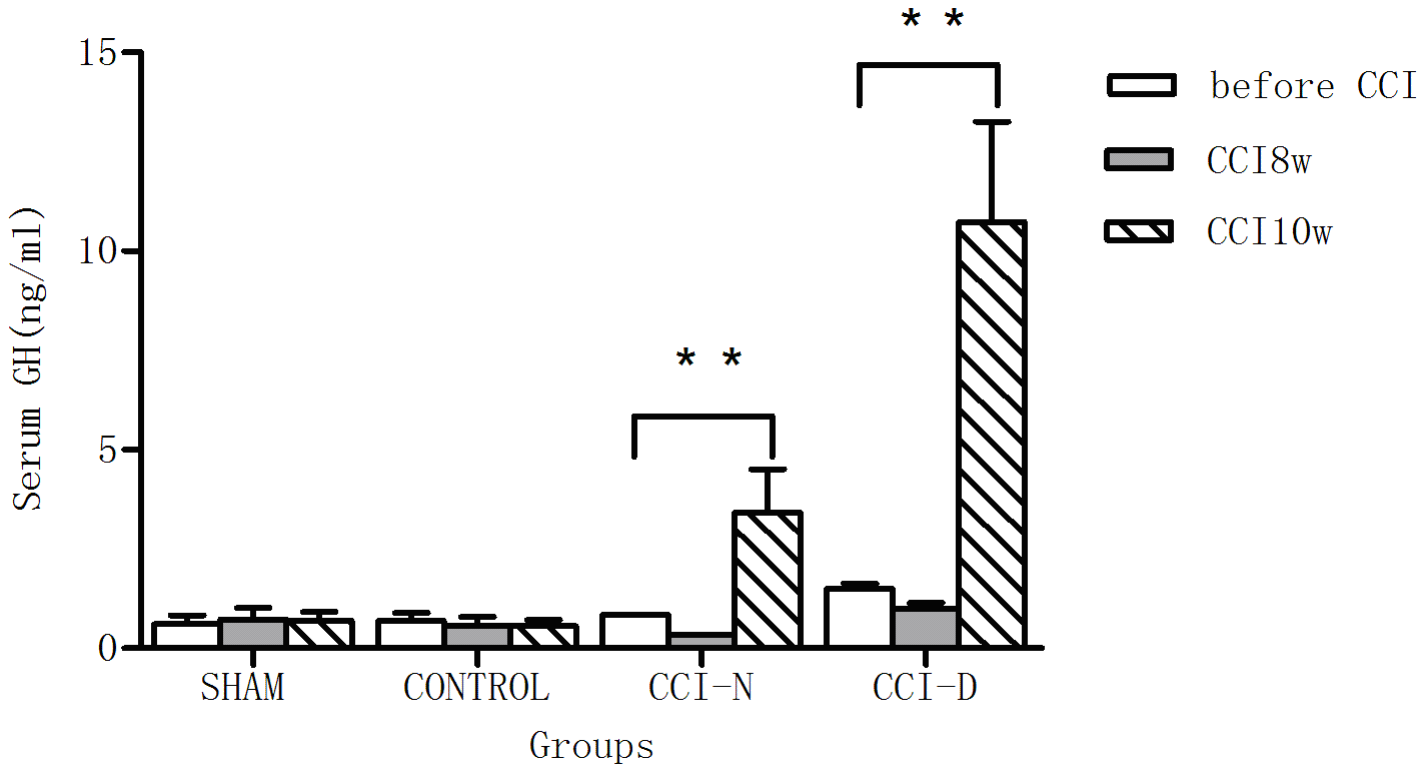
GH levels at different time points. Note: ** indicated as p<0.01 between different time points.

Fig. 1 shows that the GH level changed 8 weeks post-CCI and the effect of rhGH treatment(including 5 death and 19 GH level reduced, the death reason maybe flatulence and malnutrition). There was no significant difference in the GH level between 8 and 10 weeks post-injury (P>0.05). Whether or not the GH level was decreased, rhGH treatment increased the GH level in CCI rats (P<0.01).

### 2.2 Result of the water maze test

**Figure 2:**
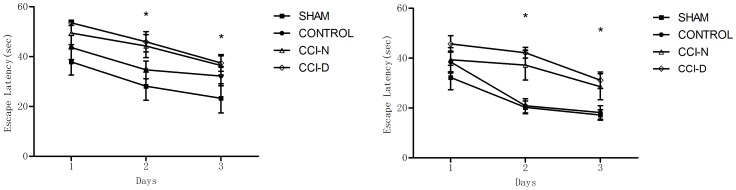
The changes of latency time. (a). 8 weeks after CCI (left). (b). 2 weeks after rhGH treatment (right). Note: *difference of P<0.05 between the four groups at the same time point.

Fig. 2(a) and 2(b) show that, at 8 weeks post-injury, there was a significant difference among the four groups (P<0.05); after 2 weeks of treatment, there was also a significant difference among the four groups (P<0.05). The latency after rhGH treatment was less than before treatment.

### 2.3 Results of spatial memory in the water maze test

**Figure 3:**
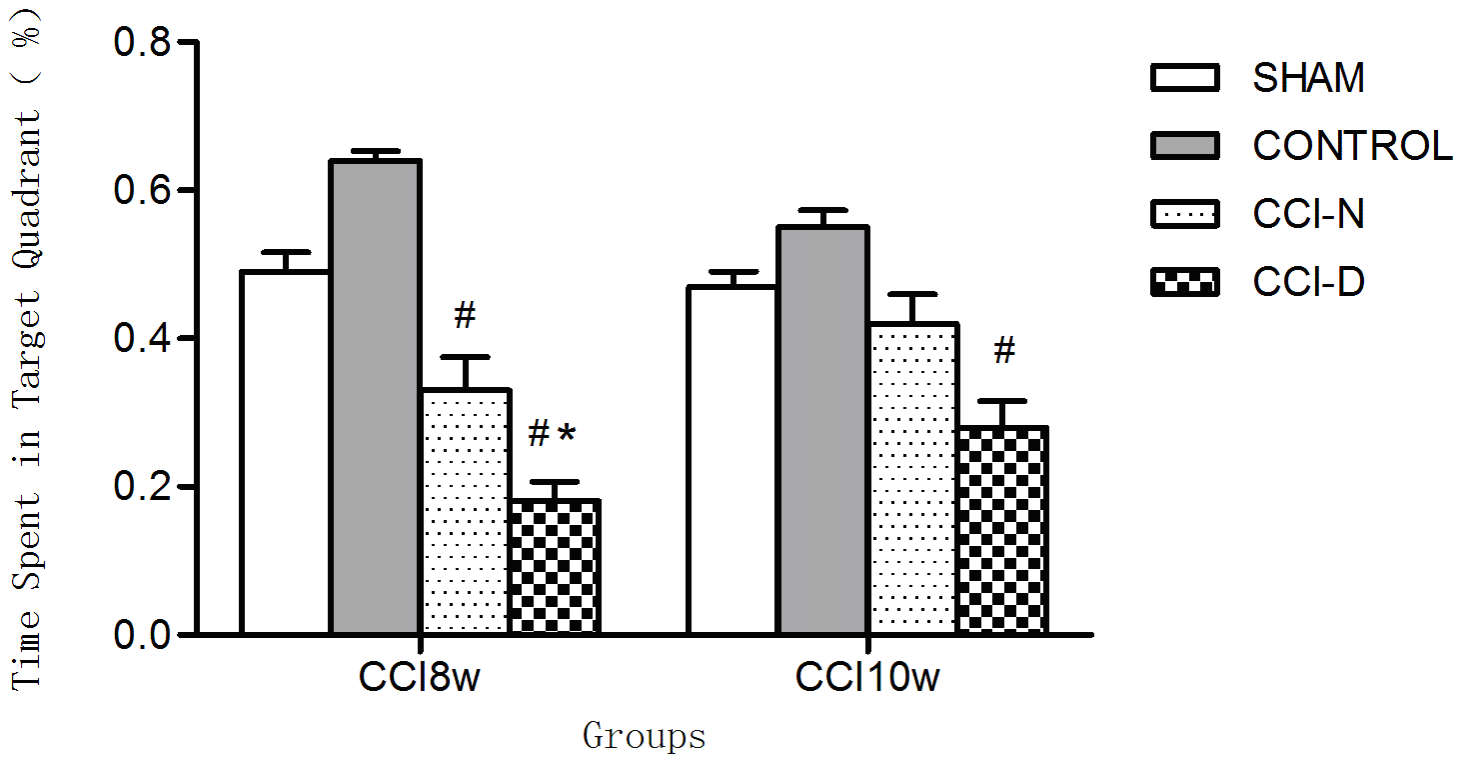
Time spent in the targeted quadrant after 2 weeks of rhGH treatment. Note: * P<0.05 compared to the sham group; # P<0.05 compared to the control group.

Shown in Fig. 3, at 8 weeks post-CCI, the time spent in the targeted quadrant was significantly different among the 4 groups (P = 0.001). After 2 weeks of treatment with rhGH, there was no significant difference between the CCI-D and CCI-N groups (p = 0.100). As shown in the bar graph, regardless of the level of GH reduction, the time spent in the targeted quadrant increased after rhGH treatment.

### 2.4 The results of novel object recognition test

**Figure 4:**
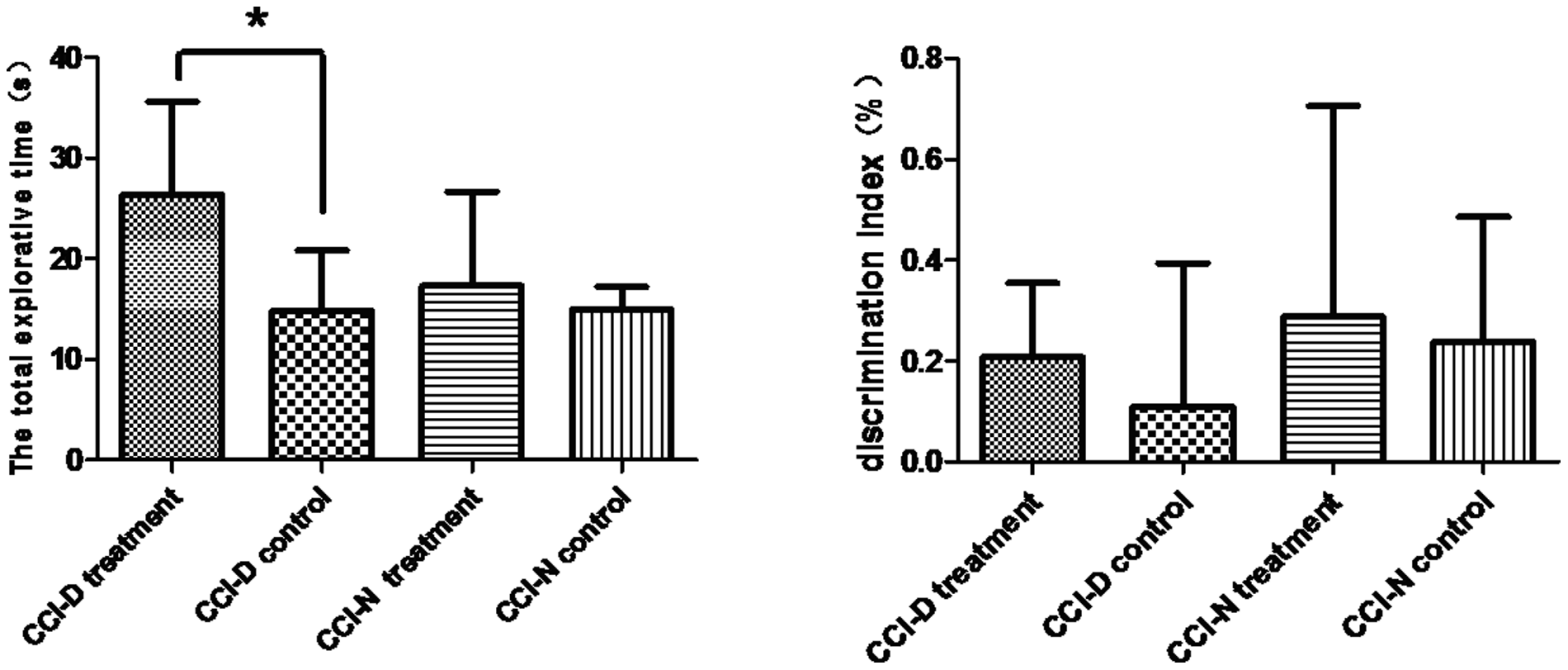
The total exploration time and discrimination index after rhGH treatment. (a) total exploration time (left). (b). discrimination index (right). Note: * P<0.05 between the two groups.

Shown in Table 2 and Fig. 4(a)(b), total exploration time in CCI-D group (rhGH treatment for 2 weeks) can be seen significant difference statistically compared with control rats. But there is no statistical significance about total exploration time in CCI-N group (rhGH treatment for two weeks) compared with control. Before and after the rhGH treatment for CCI-D and CCI-N group, the significant differences about discrimination index were not be seen.

**Table 2:** The results of novel object recognition test before rhGH treatment.

Group	Number of rats	Total exploration time (s)	DI (%)
Control	10	34.83±8.77	20.23±7.87
Sham-operated	10	30.42±3.93	15.52±3.78
CCI-N	20	**16.71±4.15** *	18.86±3.96
CCI-D	20	**17.08±3.13** *	17.71±5.41

*P<0.05 compared to control group.

### 2.5 The correlation between transmitter mRNA expression at different sites with results of water maze testing (shown in Table 3 and 4)

### 2.6 Effect of rhGH treatment on gene expression at different sites

**Figure 5:**
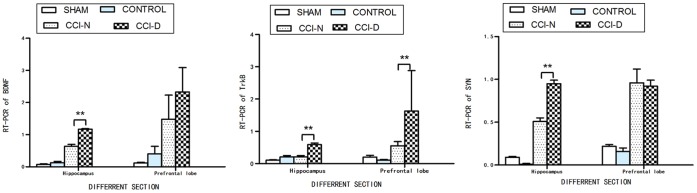
The effect on mRNA levels after rhGH treatment. (a). BDNF mRNA levels (left). (b). TrkB mRNA levels (middle). (c). SYN mRNA levels (right). Note: ** indicated as P<0.05 between the two groups.

Shown in Fig. 5(a)–(c), the results of real-time PCR demonstrated that there were significant differences in the expression of hippocampal and prefrontal Trk, BDNF, and SYN mRNA between the groups (P<0.05). Furthermore, the expression of hippocampal and prefrontal TrkB mRNA was also different between the CCI-N and CCI-D groups (p = 0.004 and 0.005, respectively). The expression of hippocampal BDNF and SYN mRNA was also significantly different between the CCI-N and CCI-D groups (p<0.05).

## Discussion

### 3.1 Effect of long-term GH level on cognitive function following TBI in rats

According to the clinical imaging data, approximately 30% of patients with TBIs develop morphologic changes in the pituitary in the acute stage [26]. Any degree of TBI can cause varying changes in pituitary hormone levels, and 3 months after the TBI, the most common is a GH deficiency [27]. A study has shown that the GH level is lower in TBI rats 8 weeks post-injury [28]. In the current study, we increased the subject sample and set the control group, and further confirmed the fact that the GH level decreased 8 weeks post-TBI, with an incidence rate of 54.28%.

In recent years, a number of clinical studies have shown that secondary hypopituitarism occurs in patients with moderate-to-severe TBIs, and the reduced GH level is associated with cognitive impairment in these patients [2], [29]–[31]. From Figure2(a), our results showed that the control and sham groups 8 weeks after the incubation period demonstrated a decreased latency time, indicating a good spatial learning ability in rats. After 8 weeks, whether or not the GH level was decreased, the latency time in the water maze test was significantly reduced in all rats. The latency time in the CCI-D group was significantly longer than the control and sham groups, indicating that reduced GH following TBI affected the spatial learning ability in TBI rats. Similarly, the CCI-D group had the shortest time in the space exploration experiment, with a significant difference between the different groups, indicating that a reduced GH level following TBI would also affect spatial memory in TBI rats. In GH-deficiency and AD models, a reduced GH level could extend the latency time in the water maze test with impaired cognitive function [6], [32]. Leon et al. [10] studied 11 cases with reduced GH levels after TBIs and showed decreased attention, executive function, memory, and mood disorders compared with TBI patients with normal GH levels. Our study was consistent with previous clinical findings showing that the GH level declined in the chronic stage of TBIs and patients with more severe cognitive dysfunction had lower GH levels [33].

### 3.2 Long-term rhGH treatment on cognitive function following TBI

In 1985, scientists in the United States synthesized rhGH, which is identical to the natural structure and function of human GH. Recent studies have shown that rhGH has some neuroprotective and angiogenic effects, thus improving cognitive function in a variety of diseases. Clinical studies have shown that patients with GH deficiency treated with subcutaneous physiologic requirement of GH can increase the GH concentrations in cerebrospinal fluid, and further improve their attention and memory. Whether or not adults or children are GH-deficient, rhGH therapy can improve cognitive function [34]–[37]. In animal experiments, it has been confirmed that both GH and GHRH can promote long-term memory in young rats, and prolong the reaction time in young and aged rats during fear subside experiments [36].

Shown in Figure 2(b), in contrast, the latency time in the two groups decreased after 10 weeks of feeding, and there was still a shorter latency time in the CCI-D group compared to the CCI-N group. After rhGH treatment, the latency time was shortened compared to that at 8 weeks and the time spent in the CCI-D group was longer than the CCI-N group. This indicated the positive effect of rhGH treatment on spatial learning function, which clearly occurred in the CCI-D group. As shown in Figure 3, whether or not the GH level is reduced, rhGH treatment increased the time spent in the targeted quadrant. This finding also indicated that rhGH therapy improved spatial memory in rats. In clinical studies it has been suggested that rhGH treatment for >1 year improves patient memory, attention, executive function, abstract thinking, and quality of life [8], [11], [38], [39]; however, these clinical studies were limited due to the small sample size and large variability between individuals, and because of the ethical limitations and clinical research. Thus, rhGH treatment is not an option for patients with normal GH levels and there are few studies regarding this clinical situation experimentally. In the current study, the effect of rhGH treatment on cognitive function not limited to TBI rats with reduced GH levels is reported for the first time.

### 3.3 Possible mechanisms underlying the effect of GH treatment on cognitive function

It has been shown that several brain structures are related to cognitive function, such as the hippocampus, striatum, basal forebrain, and neocortical structures. It has also been reported that the hippocampus, thalamus, putamen, and choroid plexus are associated with the expression of GH [40]. In addition, lesions in the hippocampus and prefrontal lobes exhibit cognitive deficits, and parietal damage does not cause aa deficit [41]. Therefore, we selected the hippocampus and prefrontal cortex for analysis in our study. Currently, the mechanism underlying the effect of GH treatment on cognitive function remains unclear. Some studies have shown that the mechanism underlying the effect of GH treatment on cognitive function is associated with a neuroprotective effect, inhibition of the inflammatory response [42], and promotion of angiogenesis [43] or promotion of cell growth via IGF-1 [44] and relevant neuroprotection [45].

### 3.4 Effect of GH on TrkB and BDNF

BDNF was extracted and purified from swine brain as decribed by Barde et al. in 1982 [15]. BDNF was shown to have a molecular weight of 13 ku, and an isoelectric point of 9.99. BDNF is mainly synthesized in the brain, and has a wide distribution in the central nervous system, especially in the cortex and hippocampus. Recent animal and clinical studies have shown that BDNF treatment after TBI has a neuroprotective effect [46]. The roles of BDNF include neutralizing high concentrations of amino acid which lead to neurotoxicity [47], down-regulation of the NMDA receptor, and inducing the expression of calcium-binding proteins to stabilize the intracellular Ca^2+^ concentration, thus reducing the generation of free radicals [48], inhibition of apoptosis [49], and reducing hypoxic-ischemic brain damage and promoting regeneration of damaged neurons. BDNF is a cerebral protection factor that contributes to the repair of brain damage and has a protective effect on neurons. BDNF can combine with the TrkB receptor to initiate autophosphorylation and activate other signaling pathways, and thus facilitates the synthesis and release of neurotransmitters involved in learning and memory, thereby playing a role in promoting learning and memory. Shown in Table 2‵3 and Figure4(b), our study showed that hippocampal and prefrontal TrkB mRNA levels were significantly different before and after rhGH therapy, with an increasing trend after rhGH treatment. In addition, the hippocampal TrkB mRNA level was negatively correlated with the water maze test score, suggesting that rhGH treatment can increase the expression of TrkB mRNA in rat brain after TBI and shortens the time spent for spatial exploration during the water maze test. Other experiments have confirmed that BDNF mRNA is correlated with cognitive function, thus the change in the gene alone has some significance. In adult TBI rats, decreased BDNF mRNA expression was correlated with the decline in cognitive function [50]. From Figure 1 and Figure 5(a), our study showed that regardless of decreased or normal GH, rhGH treatment following TBI could increase the serum GH level. Combined with genetic testing, it has been shown that exogenous rhGH replacement can also increase the expression of brain BDNF mRNA, secretion of GH, and ultimately affect the expression of BDNF and TrkB related to cognitive function in brain areas and improve cognitive function [46]. Shown in Figure 5(a) and 5(b), in our study, we also found there was a significant difference in the hippocampal mRNA levels of BDNF and TrkB between the CCI-N and CCI-D group. In the prefrontal area, only the TrkB mRNA level was significantly different between the two groups, suggesting that rhGH treatment has a more obvious effect on the regulation of neurotransmitters in the hippocampus. We observed a long-term change post-injury; although rhGH replacement therapy improved cognitive function, it lasted for only 2 weeks, which was too short for regulation of BDNF changes requiring TrkB, and also because there was a large number of inflammatory cytokines released in the prefrontal cortex, which had an effect on the expression of TrkB with a corresponding effect on BDNF expression.

The mechanism by which GH regulates BDNF to improve cognitive function may be via the basal forebrain cholinergic system to release acetylchloine (Ach) through positive feedback, affecting synaptic plasticity, adjusting learning and memory, and increasing Ach activity through enhanced phosphorylation of the NMDA receptor, regulating hippocampal synaptic transmission, and inducing long-term potentiation(LTP), thereby enhancing learning and memory.

### 3.5 The role of GH on SYN

SYN is a calcium-binding glycoprotein widely present in the presynaptic vesicle membrane, synthesized in the neuron cell body, and transmitted to the axon terminals. SYN is specifically located in the presynaptic axon terminals to the membrane vesicles and closely related to synaptic structure and function. The phosphorylation of SYN has a significant role in the neurotransmitter releasing process, which consists of a fast and efficient regulation system for neurotransmitter release. In recent years, studies have shown that synaptic change in morphology and function is one of the most important pathologic causes in learning and memory dysfunction. SYN, as a calcium-binding glycoprotein, is widely present in the membrane of presynaptic vesicles, and is closely related to synaptic structure and function. SYN modulates the release of neurotransmitters through phosphorylation and dephosphorylation, and gets involved in the import, transport, recycling, and synaptogenesis of synaptic vesicles, which plays a role in synaptic plasticity. Therefore, SYN is one of the important molecules in learning and memory processes. SYN has been the focus in a large number of studies; SYN is also involved in the release of Ca^2+^-dependent neurotransmitters, such as Ach and glutamate, and the circulation of synaptic vesicles and synaptic remodeling. SYN also affects the stability and mobility of synaptic vesicle through its redox state [51], [52]. Therefore, the amount of SYN protein may reflect the level of synaptic function, the transmission of synaptic information, and neurotransmitter release.

Brain synaptic plasticity undergoes major changes following TBI, and the synaptic reorganization plays an important role in the recovery of anatomy, physiology, and function. Shojo et al. [53] used a fluid percussion injury animal model of TBI to study the change in cortical SYN 2–30 days post-injury for mild, moderate, and severe brain injury. Shojo et al. [53]reported, with an increase in impact time and degree, cortical SYN increased, but the total SYN did not significantly change within the brain, and the increase in SYN at the lesion site was thought to be due to the inhibition of synaptic vesicles and the dysfunction of the synapse and transduction. Pyramidal neurons and synapses in hippocampal CA1 are thought to be the structural basis of learning and memory. Overseas studies have shown that the expression of SYN is increased in the model of learning and memory and the LTP phenomenon occurs in the hippocampus, which results in a 6-fold increase in SYN expression, while the expression of SYN is decreased in rats with cognitive dysfunction [54], indicating that the amount of SYN expression was positively correlated with learning and memory. We achieved the same results in our study. Previous studies have also shown that synaptic dysfunction and failure occur in the early stage of AD, and the decline in the synapse is the best pathologic basis to explain cognitive dysfunction, and SYN can regulate synaptic function [55]. Therefore, we suggest that the mechanism underlying improved cognitive function following rhGH treatment to be via SYN, which increases synaptic function and has a role in synaptic transmission and the release of the synaptic vesicle. In addition, GH can increase the release of excitoxic glutamate through SYN, maintain the LTP, improve message transmission, processing, and storage, and ultimately improve learning and memory.

Eastwood et al. [56] reported that in the dentate and parahippocampal gyri of the human brain, SYN is positively correlated with SYN mRNA, indicating that the expression of SYN is regulated by the encoded gene. Shown in Table 3,4 and Fig.5(c), our study showed that treatment with rhGH can significantly increase the SYN mRNA levels in the hippocampus and prefrontal area, and SYN mRNA is negatively correlated with water maze test scores, suggesting that rhGH treatment can increase SYN mRNA expression, which is associated with a shortened water maze navigation time. Therefore, we speculated that the improved cognitive function may be caused by up-regulating SYN mRNA. Shown in Figure 5(c), in the current study we also found a significant difference in the SYN mRNA in the hippocampus between the CCI-N and CCI-D groups. This indicated that whether or not GH is reduced, rhGH therapy can improve SYN mRNA expression. In combination with cognitive function test results, this illustrated that rhGH therapy improves cognitive function in TBI rats through up-regulation of the expression of hippocampal SYN mRNA.

**Table 3:** Correlation between prefrontal transmitter mRNA expression and water maze testing.

mRNA	Pearson coefficient	P value
BDNF	−0.458	**0.001**
TrkB	−0.974	0.224
SYN	−0.101	**0.010**

The linear correlational study showed that after rhGH treatment, the expression of prefrontal BDNF and SYN mRNA in CCI rats was significantly correlated with the results of water maze testing (r = 0.77 and 0.534, respectively; P<0.05), while the expression of prefrontal TrkB mRNA was not significantly associated with the results of water maze testing (r = 0.270; P>0.05).

**Table 4:** Correlation between the expression of hippocampal transmitter mRNA with results of water maze testing.

mRNA	Pearson coefficient	P value
BDNF	−0.458	**0.001**
TrkB	−0.066	**0.008**
SYN	−0.370	**0.001**

The linear correlational study showed that after rhGH treatment, the expression of hippocampal BDNF, TrkB, and SYN mRNA in CCI rats was significantly correlated with the results of water maze testing (r = 0.773‵0.672 and 0.783, respectively; P<0.05).

## Conclusion

Our study confirmed that GH deficiency damages spatial learning and spatial exploration abilities in TBI rats. Whether or not GH is reduced, rhGH treatment has a positive effect on cognitive function. The increased expression of BDNF and TrkB mRNA in the hippocampus and prefrontal region might be the basis for improved memory function following rhGH therapy. Changes in hippocampal SYN mRNA following rhGH therapy may also play a role in improving memory function. The experiment did not carry out the dynamic observation of GH level at different time poin, so the GH curve is still not clear, larger sample size and dynamic observation of GH level is needed; Because rhGH administration and dosage may have an impact on cognitive function, so the next experiment should thoroughly investigate the cognitive impairment in rats after TBI and the role of rhGH administration and dosage. In order to provide a theoretical basis for the clinical application of rhGH, and one day rhGH replacement therapy will be used in clinical and help more patients.
